# Prosocial motives underlie scientific censorship by scientists: A perspective and research agenda

**DOI:** 10.1073/pnas.2301642120

**Published:** 2023-11-20

**Authors:** Cory J. Clark, Lee Jussim, Komi Frey, Sean T. Stevens, Musa al-Gharbi, Karl Aquino, J. Michael Bailey, Nicole Barbaro, Roy F. Baumeister, April Bleske-Rechek, David Buss, Stephen Ceci, Marco Del Giudice, Peter H. Ditto, Joseph P. Forgas, David C. Geary, Glenn Geher, Sarah Haider, Nathan Honeycutt, Hrishikesh Joshi, Anna I. Krylov, Elizabeth Loftus, Glenn Loury, Louise Lu, Michael Macy, Chris C. Martin, John McWhorter, Geoffrey Miller, Pamela Paresky, Steven Pinker, Wilfred Reilly, Catherine Salmon, Steve Stewart-Williams, Philip E. Tetlock, Wendy M. Williams, Anne E. Wilson, Bo M. Winegard, George Yancey, William von Hippel

**Affiliations:** ^a^School of Arts and Sciences, University of Pennsylvania, Philadelphia, PA 9104; ^b^The Wharton School, University of Pennsylvania, Philadelphia, PA 9104; ^c^Department of Psychology, Rutgers University, Piscataway, NJ 08854; ^d^Research Department, Foundation for Individual Rights and Expression, Philadelphia, PA 19106; ^e^School of Communication and Journalism, Stony Brook University, Long Island, NY 11794; ^f^Marketing and Behavioral Science, University of British Columbia, Vancouver, British Columbia V6T 1Z2, Canada; ^g^Department of Psychology, Northwestern University, Evanston, IL 60208; ^h^Communications Department, Heterodox Academy, New York City, NY 10038; ^i^School of Psychology, University of Queensland, St Lucia, QLD 4072, Australia; ^j^Department of Psychology, University of Wisconsin-Eau Claire, Eau Claire, WI 54702; ^k^Department of Psychology, University of Texas at Austin, Austin, TX 78731; ^l^Department of Psychology, Cornell University, Ithaca, NY 14853; ^m^Department of Life Sciences, University of Trieste, Trieste 34128, Italy; ^n^Department of Psychological Science, University of California Irvine, California, CA 92697; ^o^School of Psychology, The University of New South Wales, Sydney NSW 2052, Australia; ^p^Department of Psychological Sciences, University of Missouri, Columbia, MO 56211; ^q^Department of Psychology, State University of New York at New Paltz, New Paltz, NY 12561; ^r^Ex-Muslims of North America, Washington D.C.; ^s^University of Arizona, Department of Philosophy, Tucson, AZ 85721; ^t^Department of Chemistry, University of Southern California, Los Angeles, CA 90089; ^u^Department of Economics, Brown University, Providence, RI 02912; ^v^Graduate School of Business, Stanford University, Stanford, CA 94305; ^w^Department of Sociology, Cornell University, Ithaca 14850, New York; ^x^Department of Information Science, Cornell University, Ithaca 14850, New York; ^y^Psychology Department, Oglethorpe University, Brookhaven, GA 30319; ^z^Center for American Studies, Columbia University, New York, NY 10027; ^aa^Department of Psychology, University of New Mexico, Albuquerque, NM 87131; ^bb^Network Contagion Research Institute, Princeton, NJ 08540; ^cc^Department of Psychology, Harvard University, Cambridge, MA 02138; ^dd^School of Criminal Justice and Political Science, Kentucky State University, Frankfort, KY 40601; ^ee^Department of Psychology, University of Redlands, Redlands, CA 92373; ^ff^School of Psychology, University of Nottingham Malaysia, Selangor Darul Ehsan, Semenyih 43500, Malaysia; ^gg^Psychology Department, Wilfrid Laurier University, Waterloo, ON N2L3C5, Canada; ^hh^ Independent; ^ii^Department of Sociology, Baylor University, Waco, TX 76798; ^jj^Research with Impact, Brisbane, Queensland 4069, Australia

**Keywords:** censorship, academic freedom, science reform, transparency, organizational behavior

## Abstract

Science is among humanity’s greatest achievements, yet scientific censorship is rarely studied empirically. We explore the social, psychological, and institutional causes and consequences of scientific censorship (defined as actions aimed at obstructing particular scientific ideas from reaching an audience for reasons other than low scientific quality). Popular narratives suggest that scientific censorship is driven by authoritarian officials with dark motives, such as dogmatism and intolerance. Our analysis suggests that scientific censorship is often driven by scientists, who are primarily motivated by self-protection, benevolence toward peer scholars, and prosocial concerns for the well-being of human social groups. This perspective helps explain both recent findings on scientific censorship and recent changes to scientific institutions, such as the use of harm-based criteria to evaluate research. We discuss unknowns surrounding the consequences of censorship and provide recommendations for improving transparency and accountability in scientific decision-making to enable the exploration of these unknowns. The benefits of censorship may sometimes outweigh costs. However, until costs and benefits are examined empirically, scholars on opposing sides of ongoing debates are left to quarrel based on competing values, assumptions, and intuitions.

The fundamental principle of science is that evidence—not authority, tradition, rhetorical eloquence, or social prestige—should triumph. This commitment makes science a radical force in society: Challenging and disrupting sacred myths, cherished beliefs, and socially desirable narratives. Consequently, science exists in tension with other institutions, occasionally provoking hostility and censorship (1). In liberal democracies, government censorship of science is rare (although see ref. 2). The greatest threats to scientific openness are often more diffuse and disguised as legitimate scientific criticism (e.g., rejection of dangerous and false information) (3).

Because scientific censorship is difficult to detect and measure, it is rarely empirically studied. Here, we discuss historical and modern evidence regarding the social, psychological, and institutional causes and consequences of scientific censorship. Our analysis suggests that censorship is often impelled by prosocial concerns (4–6) and by scientists (7). We also identify unknowns regarding scientific censorship and highlight how scientific institutions can improve transparency to facilitate the exploration of these unknowns.

## Historical Examples

Historical surveys of science often contrast a superstitious and illiberal past with enlightened modernity (8). Galileo’s defense of heliocentrism is rehashed in modern textbooks, albeit not entirely accurately. Although the Church ultimately sentenced Galileo, his persecution was driven primarily by Aristotelian professors who appealed to the Church’s authority to punish him (9). In 1591, Galileo’s contract was not renewed at University of Pisa, and after enduring hostility from peer professors, he left academia, apparently viewing it as hopelessly unscientific. In the sixteenth to eighteenth centuries, state censors (often academics themselves) revised and rejected manuscripts in a system similar to peer review (10). Some criticisms involved quality issues; others were based on fear of causing offense or of professional societies’ reactions. A 1948 survey of clinical and abnormal psychologists found that 17% of men and 25% of women wanted Kinsey’s sexuality research censored (11). Although the authors highlighted possible authoritarian motives for censorship, some psychologists cited moral concerns about vulnerable groups, e.g., “this book (Kinsey report) is already having a corrupting influence on the young, the suggestible, the weak…” (p. 287).

One might hypothesize that scientific censorship is rarer today than in the past, when science was less influential. However, the ascendancy of science does not guarantee that censorship has become a relic; one could predict the opposite. Higher stakes may create stronger incentives for censorship, especially when findings are perceived by some as potentially harmful (12).

## Types of Censorship and Censors

We define scientific censorship as actions aimed at obstructing particular scientific ideas from reaching an audience for reasons other than low scientific quality. Censorship is distinct from discrimination, if not always clearly so. Censorship targets particular ideas (regardless of their quality), whereas discrimination targets particular people (regardless of their merit). Academics have long discriminated against various types of people (e.g., women and scholars of color), a problem that has been explored by numerous scholars (13–24). Majority groups in academia influence the topics and perspectives considered worthy of study, which can cause epistemic exclusion of minority scholars and their ideas (25). More generally, scholars inadvertently suppress ideas they personally deem uninteresting or unimportant and thus unworthy of publication. This lack of interest may contribute to systemic suppression of particular ideas. Table 1 provides a taxonomy of censorship, censors, motivations, and consequences.

**Table 1.** Taxonomy of scientific censorship

Types of censorship	
Hard	Authorities (e.g., governments, universities, academic journals, professional societies) exerting power to prevent dissemination
Soft	Formal or informal social punishments or threats of them (e.g., ostracism, reputational damage) aimed at pressuring the target
Censors	
Government	Political figures and other governmental institutions
Institutions	Universities, professional societies, journals, publishers, funding agencies, and other organizations
Individuals	Peer scholars, activists, donors, reviewers, or other members of the public
Self	Scholars choosing not to pursue or disseminate their own controversial ideas
Motivations of censors	
Self-protection	Protect one’s own reputation
Self-enhancement	Elevate one’s own status as virtuous or otherwise valuable
Benevolence	Protect the target of censorship from negative consequences
Prosocial	Protect third parties from the censored content
Punitive	Control narrative and punish the target of censorship
Outcomes of censorship	
Success	Prevents censored content from reaching all or some of the intended audience
Conflict	Creates public controversy, persuading some that the content has been discredited, and others that illegitimate censorship has occurred
Backfire	Censorship attempt brings more attention or legitimacy to the content

Note. Different censorship motives are not necessarily mutually exclusive and, in some cases, may be positively related.

### Hard vs. Soft Censorship.

Hard censorship occurs when people exercise power to prevent idea dissemination. Governments and religious institutions have long censored science (26). However, journals, professional organizations, universities, and publishers—many governed by academics—also censor research, either by preventing dissemination or retracting postpublication (27–31). Soft censorship employs social punishments or threats of them (e.g., ostracism, public shaming, double standards in hirings, firings, publishing, retractions, and funding) to prevent dissemination of research. Department chairs, mentors, or peer scholars sometimes warn that particular research might damage careers, effectively discouraging it (32). Such cases might constitute “benevolent censorship,” if the goal is to protect the researcher.

### The Censors.

Worldwide, scientists have faced government suppression ranging from threats of withheld funding to job loss, prison, and even execution (33), although severe penalties have become rare. A recent set of restrictions imposed by the Hungarian government on Central European University ultimately caused the university to relocate to Austria (34). In addition, legislation across US state governments has banned teaching critical race theory (2).

A second class of censors includes institutions: universities, journals, and professional societies. Individuals backed by institutional power may censor unilaterally. Deans and department heads can withhold resources or denounce scholars who forward controversial claims. Tenure makes it difficult to fire professors but offers little protection from other punishments, and academics are increasingly nontenure track (35). Professional societies can expel, sanction, or censure members for sharing unpopular empirical claims (36) and journal editors can reject or retract controversial articles (37).

A third class exerts influence informally. Faculty members can ostracize and defame peers, pressuring them into self-censorship. Ostracism and reputational damage may seem trivial compared to historical forms of censorship, but humans value and depend on positive reputations (38), and people report a preference for various physical punishments over reputational damage (39). Even threats of denunciation are sufficient to deter scientists from pursuing unpopular conclusions they believe to be true (7, 40). Facing backlash, some scholars have retracted their own papers even when they identified no errors (41, 42). Institutions also fear reputational (and financial) damage, and so, individuals inside and outside academia can use whisper campaigns and social media to pressure institutions to censor, and wealthy donors can threaten withheld funding to do so (28). Reviewers can recommend rejection of papers or grant applications they regard as morally distasteful. Some scholars even advocate for morally motivated rejections (4, 43).

These three types of censors encourage scientists to self-censor their own controversial research (44–46). Self-censorship has been rising in the United States for decades (47), and we have little reason to expect scientists are immune to this socio-cultural trend. Nearly all US scientists report self-censoring their empirical beliefs somewhat (40, 48).

## Distinguishing Scientific Rejection from Censorship

Contemporary scientific censorship is typically the soft variety, which can be difficult to distinguish from legitimate scientific rejection. Science advances through robust criticism and rejection of ideas that have been scrutinized and contradicted by evidence (49). Papers rejected for failing to meet conventional standards have not been censored. However, many criteria that influence scientific decision-making, including novelty, interest, “fit”, and even quality are often ambiguous and subjective, which enables scholars to exaggerate flaws or make unreasonable demands to justify rejection of unpalatable findings (42, 50, 51). Calls for censorship may include claims that the research is inept, false, fringe, or “pseudoscience.” Such claims are sometimes supported with counterevidence, but many scientific conclusions coexist with some counterevidence (52). Scientific truths are built through the findings of multiple independent teams over time, a laborious process necessitated by the fact that nearly all papers have flaws and limitations. When scholars misattribute their rejection of disfavored conclusions to quality concerns that they do not consistently apply, bias and censorship are masquerading as scientific rejection.

Censorious reviewers may often be unaware when extrascientific concerns affect their scientific evaluations (53), and even when they are aware, they are unlikely to reveal these motives. Editors, reviewers, and other gatekeepers have vast, mostly unchecked freedom to render any decision provided with plausible justification. Authors have little power to object, even when decisions appear biased or incompetent.

The inherent ambiguities in peer review can also lead scholars whose work warrants rejection to believe erroneously that their work has been censored. Several scientists recently reported that they were censored for challenging mainstream views surrounding COVID-19 (54). Without access to counterfactual reality where the same methods produced different conclusions, such anecdotal accusations are difficult to confirm. Double standards are often detectable only through systematic study. For example, Ceci et al. (51) found that ethics boards were likelier to reject proposals testing discrimination against white males than otherwise identical proposals testing discrimination against women and minorities. However, boards justified their rejections with seemingly legitimate concerns (e.g., small sample size) that were not consistently applied. The potential for camouflaged censorship by decision-makers and inaccurate charges of censorship by scholars whose work warrants rejection makes identification of censorship challenging.

### Bias and Science.

People disproportionately search for (55), share (56), and remember (even falsely) preferred information (57). In addition, people are selectively skeptical of discordant information (58) and more negatively evaluate scientific methods when results are undesirable (59, 60). Similar patterns occur among scientists. For example, peer reviewers evaluate research more favorably when findings support their prior beliefs, theoretical orientations, and political views (61–63). Scientific papers describe ideological outgroup members more negatively than ingroup members (64). Scholars are likelier to reject papers ostensibly written by little-known authors than identical papers ostensibly written by prominent authors (65). In an analysis of scientific papers, 96% of statistical errors directionally supported scientists’ hypotheses, suggesting credulity among scholars toward favorable outcomes (66). In addition, a survey of *Society of Experimental Social Psychology* members revealed that perceived undesirability of an empirical finding corresponded with disbelief in that finding (67). Confirmation bias and other forms of motivated cognition (68) can fuel a self-reinforcing dynamic in which censorship and self-censorship discourage empirical challenges to prevailing conclusions, encouraging a false consensus that further discourages dissent.

Still, science is a uniquely powerful form of information gathering because it is designed to overcome biases (69). Over time, flawed ideas tend to get discarded. Myriad scientific findings, even when initially vehemently denied, were eventually accepted when evidence became overwhelming. In addition, psychology’s replication crisis has led to practices such as registered reports that reduce publication biases and improve the scientific record (70, 71). However, these practices do not address bias in science evaluations.

*Peer review* is intended to improve scientific knowledge by capitalizing on expertise. Yet, peer review itself is susceptible to bias. Editors and grant panels, often aware of well-known scientists’ inclinations, can select reviewers who share their own preferences. Because nearly all science is imperfect, peer review can obfuscate biases by cloaking selective, arbitrary, and subjective decisions in seemingly meritocratic language (72).

*Intellectual competition* can combat bias by leveraging scientists’ biases against one another. Indeed, some contend that the “dispassionate scientist” is a myth and that competing passions drive scientific progress (73). Competition encourages independent scholars to publish their most persuasive data and arguments and allows the scientific community to accept the most compelling information. This process, however, only works when scholars have competing interests and a level playing field. When scientists share preferences, competition may support systematic suppression of dissent (52).

Most modern academics are politically left-leaning (74), and so certain right-leaning perspectives are likely targets for censorship (42, 75). However, if academics were overwhelmingly right-leaning or a different ideology, opposition to that ideology would be likely targets for censorship (as when religious concerns interfered with Galileo’s scholarship). Whenever sociopolitical concerns impact evaluations of science, there is potential for systematic distortion of empirical reality.

## The Psychology of Censorship

Censorship research typically explores dark psychological underpinnings such as intolerance, authoritarianism, dogmatism, rigidity, and extremism. Authoritarianism (76, 77), on the political right and left (78, 79), is associated with censoriousness, and censorship is often attributed to desires for power and authority (11). Although citizens in liberal democracies support free speech in the abstract, they often support censorship in ideologically challenging cases (80, 81). Censorship may also signal in-group allegiances (82), as members denounce others to gain status and affirm their group’s superiority (83).

But censorship can be prosocially motivated (84). Censorious scholars often worry that research may be appropriated by malevolent actors to support harmful policies and attitudes (4). Both scholars and laypersons report that some scholarship is too dangerous to pursue, and much contemporary scientific censorship aims to protect vulnerable groups (4, 85, 86). Perceived harmfulness of information increases censoriousness among the public (3, 87), harm concerns are a central focus of content moderation on social media (88), and the more people overestimate harmful reactions to science, the more they support scientific censorship (86). People are especially censorious when they view others as susceptible to potentially harmful information (89, 90). In some contemporary Western societies, many people object to information that portrays historically disadvantaged groups unfavorably (60, 91), and academia is increasingly concerned about historically disadvantaged groups (92). Harm concerns may even cause perceptions of errors where none exist (53, 86).

Prosocial motives for censorship may explain four observations: 1) widespread public availability of scholarship coupled with expanding definitions of harm (93) has coincided with growing academic censorship (94); 2) women, who are more harm-averse and more protective of the vulnerable than men (95, 96), are more censorious (48, 77, 78); 3) although progressives are often less censorious than conservatives (86), egalitarian progressives are more censorious of information perceived to threaten historically marginalized groups (91, 97); and 4) academics in the social sciences and humanities (disciplines especially relevant to humans and social policy) are more censorious and more censored than those in STEM (98, 99).

## Censorship among Scientists

Despite the challenges of detecting censorship, recent attempts to quantify the issue have concluded that censorship motivated by harm concerns is common. Hundreds of scholars have been sanctioned for expressing controversial ideas, and the rate of sanctions has increased substantially over the past 10 y (94). Retractions of scientific articles have increased since at least 2000 (100), many for good reasons such as statistical errors, but some were at least partly motivated by harm concerns (42, 101, 102).

Surveys of US, UK, and Canadian academics have documented support for censorship (98). From 9 to 25% of academics and 43% of PhD students supported dismissal campaigns for scholars who report controversial findings, suggesting that dismissal campaigns may increase as current PhDs replace existing faculty. Many academics report willingness to discriminate against conservatives in hiring, promotions, grants, and publications, with the result that right-leaning academics self-censor more than left-leaning ones (40, 75, 99, 103).

A recent national survey of US faculty at four-year colleges and universities found the following: 1) 4 to 11% had been disciplined or threatened with discipline for teaching or research; 2) 6 to 36% supported soft punishment (condemnation, investigations) for peers who make controversial claims, with higher support among younger, more left-leaning, and female faculty; 3) 34% had been pressured by peers to avoid controversial research; 4) 25% reported being “very” or “extremely” likely to self-censor in academic publications; and 5) 91% reported being at least somewhat likely to self-censor in publications, meetings, presentations, or on social media (48).

A majority of eminent social psychologists reported that if science discovered a major genetic contribution to sex differences, widespread reporting of this finding would be bad (67). In a more recent survey, 468 US psychology professors reported that some empirically supported conclusions cannot be mentioned without punishment (40), especially those that unfavorably portray historically disadvantaged groups. A majority of these psychology professors reported some reluctance to speak openly about their empirical beliefs and feared various consequences if they were to do so. Respondents who believed taboo conclusions were true self-censored more, suggesting that professional discourse is systematically biased toward rejecting taboo conclusions. A minority of psychologists supported various punishments for scholars who reported taboo conclusions, including terminations, retractions, disinvitations, ostracism, refusing to publish their work regardless of its merits, and not hiring or promoting them. Compared to male psychologists, female psychologists were more supportive of punishments and less supportive of academic freedom, findings that have been replicated among female students and faculty (48, 98, 104–106).

**Fig. 1.**
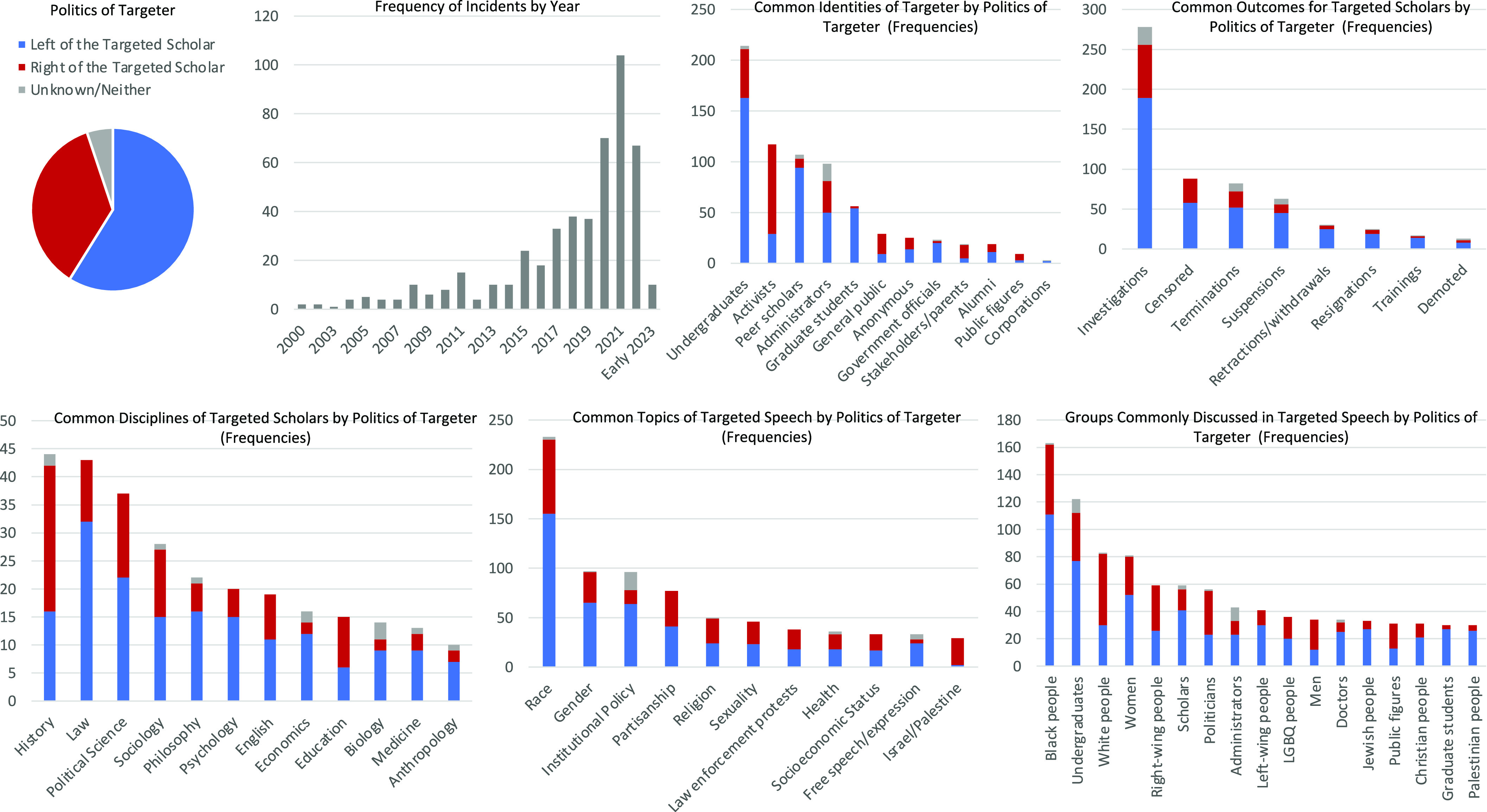
Characteristics of higher education scholars targeted for their pedagogy and/or critical inquiry between 2000 and June, 2023 (n = 486) and characteristics of their targeters.

Research on scientific censorship has often been undertaken by scientists working for nonprofits rather than by scholars publishing in peer-reviewed journals. The Foundation for Individual Rights and Expression (FIRE) has tracked 486 cases of scholars targeted specifically for their pedagogy or scholarship (94) (i.e., excluding cases of speech made outside the contexts of teaching or research) between 2000 and June, 2023 (Fig. 1). The topic of race, especially comments about Black people, triggered the most calls for censorship. Although undergraduates initiated the most censorship attempts, peer scholars, graduate students, and administrators were among the top five groups most likely to target scholars. Of 64 cases of scholars targeting peers for scholarship, nearly all involved harm concerns.

In a 2023 survey of academics in New Zealand, 53% reported that they were not free to state controversial or unpopular opinions, 48% reported that they were not free to raise differing perspectives or argue against the consensus among their colleagues, and 26% reported that they were not free to engage in the research of their choice (107). All these numbers increased from a similar survey the year before. Like scholars in the United States, New Zealand scholars felt the least comfortable discussing issues related to race, colonialism, and sex and gender.

Moral motives likely have long influenced scientific decision-making and contributed to systematic censorship of particular ideas, but journals are now explicitly endorsing moral concerns as legitimate reasons to suppress science (4). Following the publication (and retraction) of an article reporting that higher proportions of male (vs. female) senior collaborators were associated with higher post-collaboration impact for female junior authors (102, 108), *Nature Communications* released an editorial promising increased attention to potential harms (109). A subsequent *Nature* editorial stated that authors, reviewers, and editors must consider *potentially harmful implications* of research (110), and a *Nature Human Behavior* editorial stated that it might reject or retract articles that have potential to undermine the dignities of human groups (4). These policies differ from ethical concerns regarding measurable harms to participants in the process of conducting research (the purview of university ethics boards) and instead concern possible, unspecified harms that could result from dissemination of findings. In effect, editors are granting themselves vast leeway to censor high-quality research that offends their own moral sensibilities.

It may be reasonable to consider potential harms before disseminating science that poses a clear and present danger (6), when harms are extreme, tangible, and scientifically demonstrable, such as scholarship that increases risks of nuclear war, pandemics, or other existential catastrophes (111). However, the pursuit of knowledge has a strong track record of improving the human condition (112). Thus, it seems reasonable to balance knowledge risks against the costs of censorship (and resulting ignorance) by creating empirical and transparent measures of purported harms, rather than leaving censorship decisions to the intuitions and authority of small and unrepresentative editorial boards.

## Consequences of Censorship

**Fig. 2.**
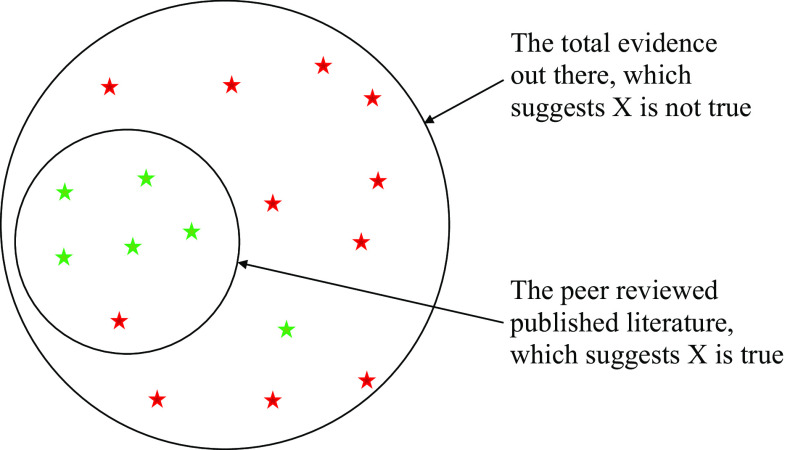
The potential epistemic consequence of scientific censorship. Green stars are evidence that X is true. Red stars are evidence that X is not true. Assume that each piece of evidence is equally weighty. Censorship that obstructs evidence against X will produce a peer-reviewed literature that concludes that X is true when most likely it is not.

There is at least one obvious cost of scientific censorship: the suppression of accurate information. Systematic censorship, and thus systematic misunderstandings, could emerge if a majority of scientists share particular preferences or prejudices that influence their scientific evaluations. Fig. 2 illustrates how the published literature could overwhelmingly indicate that X is True, even if X is more often Not True. If social processes align to discourage particular findings regardless of their validity, subsequent understandings of reality will be distorted (113), increasing the likelihood of false scientific consensus and dysfunctional interventions that waste valuable time and resources for no benefit or possibly even negative consequences (114).

Scientific censorship may also reduce public trust in science. If censorship appears ideologically motivated or causes science to promote counterproductive interventions and policies, the public may reject scientific institutions and findings (115, 116). Indeed, a recent investigation found that *Nature’s* endorsement of Biden undermined trust both in *Nature* and scientists in general (117). Loss of trust may reduce skeptics’ willingness to cooperate with scientific recommendations at crucial moments (e.g., during pandemics), causing avoidable problems for public health and safety (118, 119). A broader erosion of trust in institutions could have downstream consequences for liberalism, pluralism, and democracy.

Censorship may be particularly likely to erode trust in science in contemporary society because scientists now have other means (besides academic journals) to publicize their findings and claims of censorship. If the public routinely finds quality scholarship on blogs, social media, and online magazines by scientists who claim to have been censored, a redistribution of authority from established scientific outlets to newer, popular ones seems likely. Given the many modes of dissemination and public availability of data, proscribing certain research areas for credentialed scientists may give extremists a monopoly over sensitive research (120). Scientific censorship may also reduce trust in the scientific literature among scientists, exacerbating hostility and polarization. If particular groups of scholars feel censored by their discipline, they may leave altogether, creating a scientific monoculture that stifles progress (121).

## Unresolved Questions and Future Directions

Scientific censorship appears to be increasing (94). Potential explanations include expanding definitions of harm (93), increasing concerns about equity and inclusion in higher education (122), cohort effects (91), the growing proportion of women in science (123), increasing ideological homogeneity (74), and direct and frequent interaction between scientists and the public on social media (124, 125). However, without rigorous meta-scientific research on scientific censorship, proposed explanations are plausible hypotheses rather than empirically supported conclusions. Below, we outline changes in policies that would promote the transparency necessary to study censorship more rigorously. We also outline directions for future research that would be facilitated by cooperation from scientific institutions.

### Open Science Evaluations: An Appeal for Transparency and Accountability.

Many science journals now require high levels of transparency and accountability from their authors. Journals should be held to these same standards.

#### Peer review.

Peer review was designed to be anonymous and confidential to protect reviewers from external pressure. Confidentiality has its costs, however, including the potential to eliminate accountability and increase bias. One way to restore accountability to peer review is to request scientific journals make the review and decision-making process as open as possible. Reviews and editorial decision letters could be provided in online repositories available to all scholars (with reviewer and editor names redacted if appropriate). In addition to increasing oversight and accountability, such transparency would allow analyses of large numbers of editorial decisions that could identify potential nonscientific concerns and double standards in decision-making that mask censorship.

This approach would allow for comparisons between journals with different censorship proclivities on scientific productivity metrics, such as impact factors, replicability, and contributions to successful interventions and technologies. Scholars could make longitudinal comparisons between pioneering journals with open review (e.g., *Nature Communications*, *Royal Society Open Science*, *The EMBO Journal*, *PeerJ*, and *Collabra: Psychology*) and scope-matched journals that decline peer review transparency.

Because the goal of censorship is to prevent publication, reviews for rejected articles (and desk rejection letters) could be especially useful. To our knowledge, only *Meta-Psychology* shares peer reviews for rejected manuscripts. To minimize negative consequences for scholars, journals could give scholars the option to make reviews for their rejected articles public (immediately or after an embargo). Some authors may decline, but others—especially those who believe their work was treated unfairly—may accept. Although imperfect, such a policy would increase accountability for reviewers and editors and facilitate the study of both scientific decision-making and censorship.

Professional societies could make available the submissions, reviews, and acceptance/rejection decisions for their conferences (perhaps with identities redacted). Because conferences attract many submissions, this policy could rapidly provide scholars with large datasets to test for biases in acceptance and rejection decisions. To enhance accuracy and completeness, willingness to include one’s submission and the resultant outcome in a publicly available dataset could be a prerequisite for conference submissions. Currently, these information-rich datasets are lost year after year.

#### Auditing academia.

Just as scholars have long conducted audit studies of organizations for biases and discrimination (126), scholars could audit scientific journals and institutions for procedural unfairness that prioritizes extraneous factors (such as social desirability of research conclusions or author identity characteristics) above research quality. Scholars could submit abstracts, manuscripts, or presubmission inquiries with identical methods but manipulated results to numerous journals and conferences to test if one set of findings is rejected more often than another and to explore biased rejection decisions such as harsher critiques of (identical) methods. Journals and professional societies should consent to such audits, especially given the frequency with which they report similar audits of other institutions in their journals and at their conferences. This approach would enable scholars to study what gets censored and how censorship is justified, while also increasing accountability among decision-makers.

Scholars could also conduct large-scale surveys of scientists to evaluate journals for perceived procedural fairness. Some journals (e.g., PNAS) already survey submitters on relevant questions, such as submitters’ satisfaction with the reasons provided for rejection, but to our knowledge, no journals make these data publicly available. Allowing submitters to evaluate reviewers, editors, and journals and sharing these data publicly would 1) enable empirical analysis of perceived procedural unfairness and censorship, 2) provide submitters some power to push back against unfairness, 3) increase accountability among decision-makers, and 4) allow submitters to make better decisions about where to submit their future manuscripts.

Transparent peer review and academic audits would also help identify a related problem: lax standards for desirable findings. Just as scholars hold unpalatable findings to higher standards as a mode of suppression, so too they hold weaker standards for socially desirable (82) or seemingly important papers (127). Ironically, such double standards can create a corpus of substandard research on the most critical topics. Insofar as lax standards for one conclusion indicate stricter standards for opposing conclusions (and vice versa), auditing for one problem also facilitates discovery of the other.

#### Creating competition.

Audits and evaluations of academic journals would help facilitate competition among science journals. Currently, journal reputations rest largely on impact factors (essentially, the frequency of citations of published papers), but this likely perpetuates a Matthew Effect (128), in which journals with high impact factors attract more attention, increasing awareness of their published articles and the likelihood of citation. Metrics of editorial practice quality, fairness, commitment to truth, and sociopolitical independence would allow newer but better journals to compete, which might further inspire the creation of new journals, creating more data for comparisons between journals with different values and approaches. Similarly, scholars could compare research quality metrics between peer-reviewed journals and preprint servers to test whether the high costs of peer review (in time, money, and research delays) are producing higher benefits. This could inspire the creation of new platforms, such as low-curation journals or servers that require 1) empirical data, 2) preregistration, and 3) open methods, code, and data, but no additional hoops to jump through (e.g., convincing narratives about novelty, perfect packages of statistically significant results). Scholars could then test whether the arduous peer review process produces higher-quality science than these low-cost alternatives.

#### Retraction.

All serious calls for and considerations of retractions of published scientific papers could be documented by each outlet in a dataset shared with scholars. Scholars could code the scientific concerns raised (e.g., instruments, operationalizations, samples, analysis decisions, statistical errors, and data fraud), as well as any nonscientific concerns (e.g., moral concerns about implications or applications), in which concerns were deemed legitimate by the journal, and which contributed to a retraction decision. Such data would allow scholars to detect hidden censorship via inconsistencies in retraction for articles with similar flaws in the same journals and to test whether nonscientific concerns (such as moral or political concerns) predict the use of double standards. These data might also illuminate whether and how editors disguise nonscientific concerns as scientific ones in their retraction explanations. Scholars could also code for whether alleged harms have any empirical basis or merely reflect untested assumptions.

### Clarifying Tradeoffs and Investigating Alternatives.

Although concerns about potential future harms are a common justification for scientific censorship, few studies have examined the veracity of harm concerns. How likely, extensive, and imminent is the harm? Do experts agree on the likelihood and range of magnitudes? Do scholars from different identity or ideological groups hold different harm estimates? Some evidence suggests that harmful outcomes of research are systematically overestimated and helpful outcomes systematically underestimated (86). To test whether scientists and editors also overestimate scientific harms, their expectations about scientific consequences could be compared to eventual outcomes. Forecasting tournaments on the likely harms of controversial research could 1) test whether scientists tend to overpredict harms and 2) identify people adept at predicting realized scientific harms (129). Analyses could also use archival data. In cases when harm concerns were raised, what harm actually occurred? Is censorship the only way to minimize harm risks or are there other, potentially more effective, strategies? How often does censorship cause harm by encouraging conspiracy theories and reducing trust in science?

Scholars should empirically test the costs and benefits of censorship against the costs and benefits of alternatives. They could compare the consequences of retracting an inflammatory paper to 1) publishing commentaries and replies, 2) publishing opinion pieces about the possible applications and implications of the findings, or 3) simply allowing it to remain published and letting science carry on. Which approach inspires more and better research? Which approach is more likely to undermine the reputation of science? Which approach minimizes harm and maximizes benefits? Given ongoing controversies surrounding retraction norms, an adversarial collaboration (including both proponents and opponents of harm-based retractions) might be the most productive and persuasive approach to these research questions (52, 130).

Analysis of purported harms should be a subject of investigation within all scientific disciplines that emphasize harm risks in their norms and policies regarding acceptable scholarship. Such analyses are practically nonexistent in the behavioral sciences, where harm concerns currently influence policy (4).

## Conclusion

We have more questions than we have answers. Although many members of our research team are concerned about growing censoriousness in science, there is great diversity of opinion among us about whether and where scholars should “draw the line” on inquiry. We all agree, however, that the scientific community would be better situated to resolve these debates, if—instead of arguing in circles based on conflicting intuitions—we spent our time collecting relevant data. It is possible that there are some instances in which censoring science promotes the greater good, but we cannot know that until we have better science on scientific censorship.

## Data Availability

Previously published data were used for this work (https://www.thefire.org/research/publications/miscellaneous-publications/scholars-under-fire/) (94).
